# Case of a Wound in the Œsophagus, Accompanied with Diseased Lungs

**Published:** 1816-05

**Authors:** N. Bruce

**Affiliations:** Surgeon to the Forces


### JOURNAL AND REVIEW.

VOL. I.] MAY, 1816. [NO. 5.

### PART I.

### ORIGINAL COMMUNICATIONS.

### *Case of a Wound in the Œsophagus, accompanied with diseased Lungs.*

Communicated by N. BRUCE, Esq. Surgeon to the Forces.

TROI LUCE CELESTIN, private in the 21st Regiment of the Line, in the late army of Napoleon Buonaparte, received the point of a broad sword in the neck, during a charge of the British cavalry, which took place in the battle near *Waterloo*, on the 18th of June, 1815. The weapon entered the tendinous hollow immediately above the head of the sternum; and passing inwards, and a little to the left, appeared to have penetrated both the trachea and œsophagus. In consequence of this wound, and another received from a sabre, on the forehead, he was one of the many victims unable to escape from the field, and was a short time afterwards conveyed to the Gendarmerie Hospital at Brussels, where he received all the aid and attention that could be afforded by the Surgeons of the British army. An attempt was made to introduce food into the stomach by means of a flexible tube passed through the pharynx, beyond the wound in the œsophagus, with a view to prevent the escape of victuals by the wound, which would otherwise happen during deglutition; as well as to permit the necessary applications to be made for closing the external aperture. But this instrument produced such a violent effect upon the patient for a considerable time after its introduction, that no argument or entreaty could ever after induce him to submit to a renewal of the experiment. As he complained of the application of pressure on the part, the wound was dressed in such a manner as not entirely to prevent the escape of aliment, on the presumption that, the trachea being penetrated, the food and drink, if denied a passage externally, might find their way into the wind-pipe.

The following were the symptoms of this man's case, on the 12th of July, twenty-four days after his receiving the wound. He was greatly emaciated, his pulse much accelerated. He was harassed by a frequent cough, and an expulsion of purulent and mucous matters, especially when in the erect posture, both through the wound and by the mouth, particularly by the former. A great portion of his food escaped externally, and the bandages were continually soaked through, partly with the drink and liquid aliments which he had taken by the mouth, and partly with purulent matter. He ate with a good, or rather a voracious appetite; but his spirits were, at all times, exceedingly depressed, his temper fretful, and he seemed to have abandoned himself to despair. His strength gradually declined; he had become extremely emaciated; and he died, as was believed, of inanition, on the 30th of July.

### Dissection.

On examining the course of the wound, after death, the canal of the œsophagus was found penetrated, as may be supposed; but the opening into that tube was of more inconsiderable size than might have been expected. No wound could be discovered in the channel of the trachea, nor any trace of one. Both lobes of the lungs were studded with tubercles, and contained much purulent matter, attended with the other morbid appearances usually observed in subjects who had died of pulmonary disease; presenting, in short, that disorganization of structure, which excites our surprise that the functions of life, and of respiration, could have been sustained so long.

### Remarks.

The above man was visited by most of the medical officers of the army at Brussels, as well as by medical strangers who happened to be in that city, and was considered a hopeless case, *ab initio*. Judging from the escape of food, the exactly central direction of the wound, and the obvious circumstance of air passing through that orifice, especially during coughing, no one doubted that this man had suffered a compound injury of both trachea and œsophagus; and, in consequence of this universal belief, it was deemed perilous to apply effectual means to shut up the aperture in his neck.

This man's death may have been owing to two causes, either of which was singly adequate to produce this effect: viz. inanition; and the morbid state of the lungs, as discovered after death. We have undisputed authority for believing that wounds of the œsophagus are not always fatal:* and, as we know that life may be supported for a very long time with a very trifling portion of nutriment, we may, with greater probability, ascribe the fatal termination of this man's case to pulmonary disease, as indicated by the constant attendants of severe cough, purulent expectoration, and quickness of the pulse; which symptoms are to be regarded, almost exclusively, as symptomatic of the morbid condition of the lungs. Add to this, that no other trace of disease, or of previous inflammation, was discoverable in those parts lying between the lungs and the seat of the wound. Does this absence of continuous disease shew that the pulmonic disorganization began, and went through its course, totally independent of the wound? Did not the infliction of this injury, at any rate, materially accelerate the otherwise mortal event, by preventing the introduction of sufficient nourishment? The cause of death is, perhaps, pretty satisfactorily accounted for, from a review of the morbid appearances alone. If, however, it can be supposed, according to the general belief entertained at the time, that the sword, which inflicted the hurt in the gullet, had also, in its passage, wounded the tender canal of the windpipe, though ever so slightly, we might thence reasonably conclude, that, in addition to the pain of this last injury, some portion of the swallowed aliment, either fluid or not, would have found its way into that tube, and thence descended into the lungs; producing excessive irritation, severe cough, and all the subsequent mischief brought to view by the dissection. This, however, is only mentioned as a possible occurrence; which, if we admit, we know that sub-

---

* Parey, Garengeot, De La Faye, Schenkius, Bell, &c.

stances happening to drop into the trachea, during deglutition, will sometimes produce serious pulmonic disease,* and even death. But the result of the dissection does not admit of such an explanation; and some other circumstances in this case being wanting to render this idea probable, the supposition that the trachea was wounded, is thus entirely groundless. But whether the morbid alterations discovered in the lungs of this man are to be considered as independent of, or consequent to, the infliction of the wound, is immaterial, if we believe that such a diseased state of the pulmonary organs was incompatible with the longer continuance of life.

The practical lesson deducible from the detail and record of fatal cases is, more or less, instructive. This was, most probably, a case of wounded œsophagus occurring in a subject previously labouring under a pulmonic disease of a serious or fatal tendency. To have afforded a chance of success, it would have been necessary in the incipient stage to combat the pulmonary complaint by more early and more effectual measures than were, perhaps, practicable in this case. Wounds of the œsophagus, except when perpetrated by the determined suicide, are certainly rare; and, as they can hardly be inflicted without concomitant injury to the trachea, or to some of the large vessels in the neck, they may be considered as usually productive of immediate danger, independently of the consequent hazard of wounding a part of such importance to the due nutrition of the body as the gullet. This instance, however, shews, at least, that a simple wound of this canal is not speedily fatal: and, if it were possible, in such cases, to preserve the patient's life by the introduction of sufficient sustenance through a tube, or by clysters, as has been successfully done in cases of dysphagia, it is said, for the space of two or three months, the aperture might have time to close, and the wound be thus, in a great measure, stripped of its danger. It may be proba-

* An instance of this occurred in the month of May, 1815.—Mrs. Howes, the wife of a serjeant, stationed at Sandhurst, Berks, in swallowing a morsel of roasted meat, suffered a small piece of coal-cinder attached to it to drop into the trachea, whence it fell into the bronchia on the right side. A dangerous attack of peripneumony was the immediate consequence, which was treated in the usual manner, and had nearly proved fatal. At the end of seventeen days, a piece of cinder, of the size of a small cherry stone, was coughed up, coated with very fœtid pus, its expulsion being followed with a gradual and more decided abatement of the remaining symptoms, and the ultimate recovery of the patient.

able that in this patient the diseased state of the lungs, and morbid tenderness of the trachea, had some share in rendering the contact of the introduced tube so intolerable to the pharynx and œsophagus, by a certain sympathy or irritability of these parts; and that the wound, which might have otherwise closed, was thus kept constantly open and unquiet, by the expulsion of food and matter, and by the distress of an incessant cough.

---

